# What Determines Habitat Quality for a Declining Woodland Bird in a Fragmented Environment: The Grey-Crowned Babbler *Pomatostomus temporalis* in South-Eastern Australia?

**DOI:** 10.1371/journal.pone.0130738

**Published:** 2015-06-22

**Authors:** Kate P. Stevens, Greg J. Holland, Rohan H. Clarke, Raylene Cooke, Andrew F. Bennett

**Affiliations:** 1 School of Life and Environmental Sciences, Deakin University, Melbourne, Victoria, Australia; 2 School of Biological Sciences, Monash University, Melbourne, Victoria, Australia; 3 Department of Ecology, Environment and Evolution, La Trobe University, Melbourne, Victoria, Australia; Universidad de Granada, SPAIN

## Abstract

Understanding what constitutes high quality habitat is crucial for the conservation of species, especially those threatened with extinction. Habitat quality frequently is inferred by comparing the attributes of sites where a species is present with those where it is absent. However, species presence may not always indicate high quality habitat. Demographic parameters are likely to provide a more biologically relevant measure of quality, including a species’ ability to successfully reproduce. We examined factors believed to influence territory quality for the grey-crowned babbler (*Pomatostomus temporalis*), a cooperatively breeding woodland bird that has experienced major range contraction and population decline in south-eastern Australia. Across three broad regions, we identified active territories and determined the presence of fledglings and the size of family groups, as surrogates of territory quality. These measures were modelled in relation to habitat attributes within territories, the extent of surrounding wooded vegetation, isolation from neighbouring groups, and the size of the neighbourhood population. Fledgling presence was strongly positively associated with group size, indicating that helpers enhance breeding success. Surprisingly, no other territory or landscape-scale variables predicted territory quality, as inferred from either breeding success or group size. Relationships between group size and environmental variables may be obscured by longer-term dynamics in group size. Variation in biotic interactions, notably competition from the noisy miner (*Manorina melanocephala*), also may contribute. Conservation actions that enhance the number and size of family groups will contribute towards reversing declines of this species. Despite associated challenges, demographic studies have potential to identify mechanistic processes that underpin population performance; critical knowledge for effective conservation management.

## Introduction

Effective conservation requires an understanding of what constitutes suitable habitat for species of concern [1–3]. Suitable habitat provides resources needed for survival and reproduction, including food, shelter and nesting sites. The availability of such resources typically varies spatially in response to broad environmental gradients [4,5]. Studies that investigate the habitat requirements of species often compare ‘suitable habitat’ (species present) with ‘unsuitable habitat’ (species absent). Such presence/absence studies form the basis of most species distribution models [6,7]. However, the presence of a species at a location does not necessarily indicate habitat of high quality [8–10]. For example, population ‘sinks’ represent areas of habitat where individuals of a species occur but reproductive output falls below the threshold required for a self-sustaining population [11].

Anthropogenic land-use and landscape change also profoundly affect the availability and quality of habitat for many species [12–14] and act to amplify the variation in habitat quality associated with environmental gradients. Consequently, conservation management is likely to be more effective when it is possible to move beyond presence/absence comparisons and focus on more biologically relevant indicators of habitat quality. Demographic parameters, such as population size and reproductive output, represent two such indicators. Comparing demographic parameters across spatially separated populations will likely provide valuable insights into relative population performance, which in turn can be used to infer habitat quality [10]. Reducing the ambiguity of what constitutes high quality habitat for species will become increasingly important as more areas are subjected to human-induced disturbance [12].

Comparing population demographic parameters across broad spatial scales is considerably more challenging and time consuming than conducting presence/absence studies, explaining why studies of population demography are comparatively few. In south-eastern Australia, the habitat requirements of the grey-crowned babbler (*Pomatostomus temporalis*), a threatened species of woodland bird, have been assessed using presence/absence studies [15–17]. In an effort to shift beyond a framework of ‘suitable’ and ‘unsuitable’ habitat to more refined measures of habitat quality, we focus on demographic parameters of this species at occupied sites only. We use the presence of fledglings and family group size as surrogates for territory quality, because both display a strong positive association with reproductive success in this species [18,19]. We examine several hypotheses concerning factors that influence the quality of territories occupied by family groups of the grey-crowned babbler. Specifically we test whether reproductive capacity and group size are influenced by: 1) habitat structure and complexity within the territory (used as surrogates for local resource availability e.g. food, shelter, nest sites); 2) the extent of wooded vegetation surrounding the territory (a measure of total habitat available to each group); 3) the degree of isolation from nearby territories (used as a surrogate for habitat fragmentation and exchange of individuals between groups); and 4) the size of the neighbourhood population (used as a surrogate for habitat quality/availability at a broader landscape-level).

## Methods

### 2.1 Ethics Statement

This research was conducted under Deakin University Animal Welfare Committee approval A66-2009; Australian Bird and Bat Banding Scheme authority 1762, and Department of Sustainability and Environment (Victoria) bird banding and research permit 10005380.

### 2.2 Study species

The grey-crowned babbler is a cooperatively breeding woodland bird which lives in social groups consisting of a dominant breeding pair assisted by ‘helpers’ (usually previous offspring) [18,20]. Reproduction occurs over an extended season from June to March/April. Grey-crowned babblers feed on invertebrates taken at ground-level and also from the trunks and foliage of trees and shrubs. They construct numerous large (~40–50 cm) communal roost nests in their territory. At least one brood nest is constructed per breeding season for use by a breeding female [21]. The species is widespread in northern and eastern Australia, but has declined markedly in the southern part of its range where there has been extensive loss, fragmentation, and degradation of native vegetation [22,23]. In the south, the grey-crowned babbler persists in remnant woodland patches within agricultural landscapes, most often characterised as roadside vegetation separated by unsuitable areas of cleared farmland [23]. These remnants are some of the last vestiges of once widespread and connected woodland ecosystems of southern Australia [24,25]. As a result, the grey-crowned babbler is now listed as threatened in two states [26,27].

### 2.3 Study area

**Fig 1:**
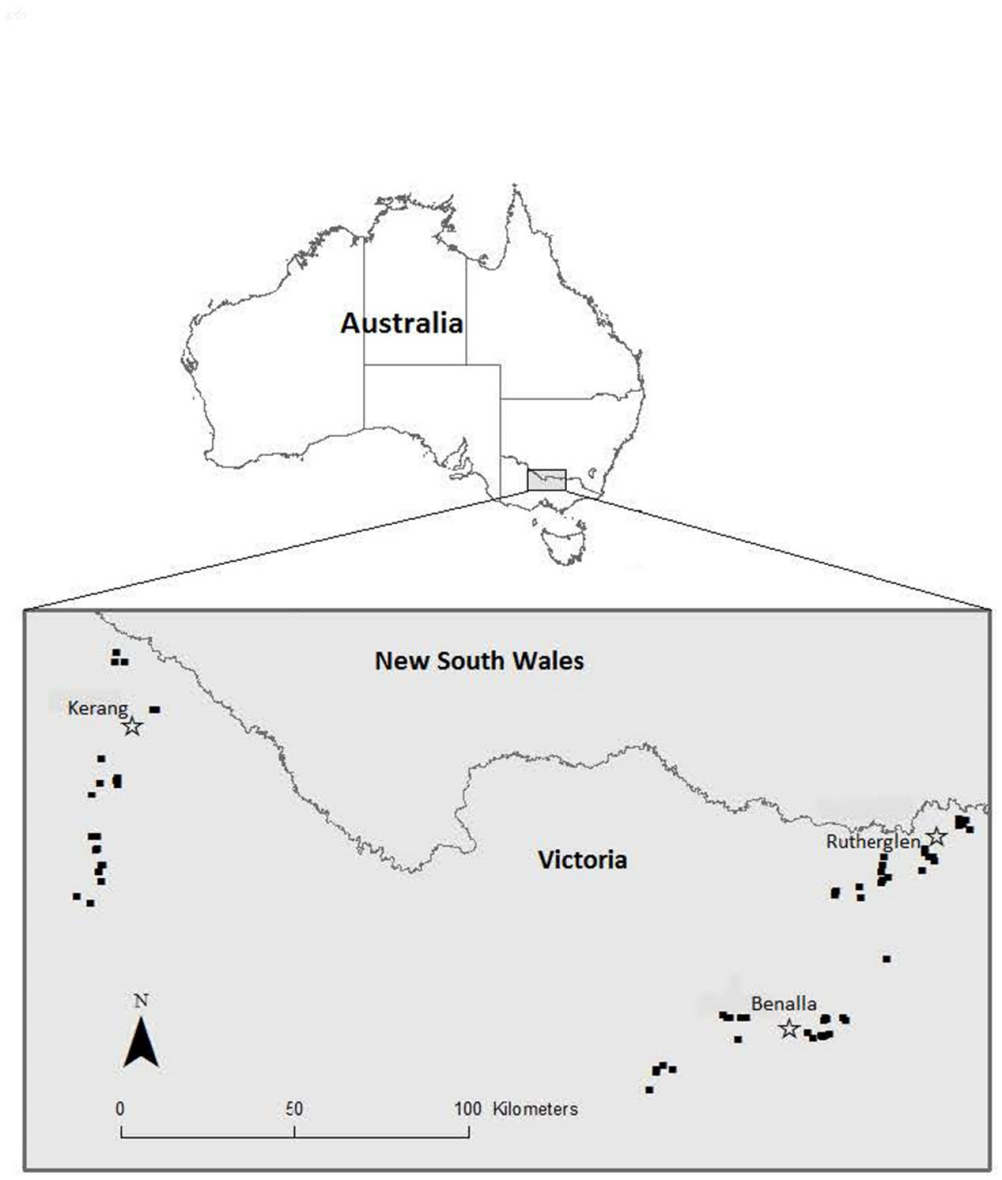
Study area in south-eastern Australia centred on three distinct regions: west (Kerang), south-east (Benalla), and north-east (Rutherglen). Grey shading = study area, open stars = towns, black dots = site locations.

The study area encompasses ~22,250 km^2^ in northern Victoria, Australia, stratified across three regions: west (centred on Kerang, 36.12°S, 143.72°E), south-east (centred on Benalla, 36.55°S, 145.98°E) and north-east (centred on Rutherglen, 36.06°S, 146.46°E) (Fig 1). These three regions support the largest populations of the grey-crowned babbler in Victoria. A gradient in mean annual rainfall occurs across this region (Kerang: 387 mm; Benalla: 651 mm; Rutherglen: 588 mm) [28]. Above average rainfall occurred in 2010 and 2011, and atypical flooding events occurred in early 2011, with large areas in the west affected by floodwaters for several months.

Native vegetation in the study area consists primarily of eucalypt woodlands that vary in composition according to soil type and rainfall. Since the mid-1800’s, some 85% of native vegetation within this area has been cleared for agriculture [29], with little vegetation remaining in its natural state. In the west, woodlands are dominated by black box (*Eucalyptus largiflorens*) with a sparse understorey of flood-tolerant vegetation such as lignum (*Muehlenbeckia florulenta*) in wetter sites, and *Acacia* spp. in drier areas. In both the south-east and north-east regions, woodland remnants are dominated by single-species stands of grey box (*E*. *microcarpa*), with smaller areas of other single or mixed-eucalypt species. Mid-storey vegetation consists of shrubs such as gold-dust wattle (*Acacia acinacea*) and golden wattle (*A*. *pycnantha*). All regions contain introduced grasses and some introduced mid-storey species such as boxthorn (*Lycium ferocissimum)* and Peruvian peppercorn (*Schinus molle*).

### 2.4 Site selection

Study sites (i.e., occupied territories) were selected based on the known occurrence of grey-crowned babblers [30,31]. Sites were stratified by region (west, south-east, north-east), with 24 territories in each (*n* = 72 territories in total, representing 3.6% of known grey-crowned babbler territories in Victoria) (S1 Table). In each region, territories were selected within both large (>30 groups) and small (≤30 groups) neighbourhood populations, with neighbourhoods separated by ≥5 km from other identified neighbourhoods or known groups. Territories were further stratified according to distance to the nearest adjacent territory (near <1 km, *n* = 47; far ≥1 km, *n* = 25). Call playback was employed to confirm the presence of grey-crowned babblers at all sites. An on-ground search using call playback was conducted in areas of suitable habitat within a 2 km radius of each study territory to determine distance to adjacent groups.

Territory size and configuration differed. Most territories (53%) were located in linear strips of roadside vegetation, 40–60 m in width. Thirty-three per cent were in small patches <5 ha, consisting of remnant vegetation, or revegetated linear strips within farmland alongside roadside vegetation. The remaining 14% of territories were in native woodland patches >5 ha, two of these incorporating private gardens which abutted large woodland patches.

### 2.5 Bird surveys

Surveys of grey-crowned babblers spanned an entire breeding season, June 2010 to April 2011 (except for two territories that were surveyed in the following 2011/12 breeding season). The breeding female of each territory displays reproductive behaviour several weeks before egg laying. A nesting cycle typically results in 2–4 eggs being laid and surviving hatchlings generally fledge after ~8 weeks [18]. Breeding success was therefore measured by visiting every territory at approximately eight week intervals over the entire breeding season, to determine whether recently fledged young were present (S1 Table). In this way, we accounted for multi-brooding events within a single season. Although fledglings may not have been recorded on some visits at some sites due to predation, we considered predation of all fledged offspring at a site over an entire breeding season unlikely. Recently fledged young were identified from adults by their small size, obvious grey ear coverts, dark brown eyes and begging behaviour; characteristics retained up to 0.5 years of age [32]. Mean group size (S1 Table) was determined from censuses recorded during each visit.

### 2.6 Vegetation surveys

Vegetation and habitat features were assessed at each site in each of three quadrats (50 X 20 m): one was located at an active roost nest, while the remaining two were located at points where grey-crowned babblers were observed foraging. This ensured that vegetation assessments were centred on active areas of each territory. Data collected in each quadrat included the number of shrubs >1 m height, percent cover of lignum, number of logs >30 cm diameter, number of stumps, and the number and species of live trees classified into size classes (<10, 10–30, 30–60, 60–90 and >90 cm diameter at breast height (DBH)). The quadrat midline provided a 50 m transect along which additional data were collected. A measuring pole was placed perpendicular to the ground at 5 m intervals along the transect (*n* = 11 points). Ground substrates that the pole touched were recorded (i.e. short grass <10 cm, herbs, leaf litter and bare ground), as was vegetation touching the pole in each 10 cm height class from 10–100 cm (i.e. long grass >10 cm and shrubs). The habitat attributes of each territory were then derived by calculating the mean value from the three quadrats.

### 2.7 Regional habitat comparisons

We used nonmetric multi-dimensional scaling (nMDS) ordination to compare the vegetation structure at sites between regions, using a Bray-Curtis measure of similarity, implemented in PRIMER [33]. We tested whether variation in vegetation structure between regions was greater than variation within regions using analysis of similarity (ANOSIM), and compared structural variation within sites for each region using SIMPER [34].

### 2.8 Variable selection for modelling

We used two dependent variables to investigate the response of the grey-crowned babbler to site and landscape characteristics: (1) breeding success of groups, as indicated by fledgling occurrence; and (2) group size (averaged across three surveys). A set of explanatory variables was derived to represent variation in habitat features across study territories. Direct measurement of resource availability (e.g. ground and bark invertebrates which represent the bulk of the grey-crowned babbler diet) is not logistically feasible across an extensive study area such as that used here. Instead, data were obtained for local habitat characteristics considered to be reliable surrogates of resource availability [35]. From the habitat data available, litter cover and the number of shrubs, large trees (>60 cm DBH), stumps and large logs (≥30 cm diameter) were selected for inclusion in models (Table 1). These variables were considered reliable surrogates for key resources including food, foraging substrates, and shelter/nesting sites [16,17,20]. A further four explanatory variables were included due to their likely influence on the dependent variables at the landscape-scale (Table 1). Local tree cover was the area (ha) of wooded cover (native wooded vegetation) within a 300 m radius of each territory, and represents the amount of habitat available to each study group. Territory isolation was represented by two variables: the average distance to neighbouring territories, and the area (ha) of wooded cover within a 1 km radius of territories. These variables represent the degree of local habitat fragmentation and act as surrogates for exchange of individuals between groups. Finally, local neighbourhood size (large: >30 groups; small: ≤30 groups) was included as a categorical variable to represent habitat quality/availability at a broader landscape-level. The last four variables are important to consider since they represent habitat availability and fragmentation; factors considered likely to have a strong influence on species with complex social systems such as cooperatively-breeding birds [36].

**Table 1:** Explanatory variables for models of breeding success and group size for the grey-crowned babbler (*Pomatostomus temporalis*).

Variable	Type	Range	Mean	Group size analysis	Breeding success analysis	Description
Leaf litter	Continuous	0.23–0.96	0.67	Yes	Yes	Average percent cover of leaf litter for three quadrats (surrogate for availability of food and foraging substrates)
Trees	Continuous	0–10	2.70	Yes	Yes	Total number of trees with trunks >60 cm DBH from three quadrats (surrogate for availability of food and foraging substrates)
Shrubs	Continuous	0–344	49.25	Yes	Yes	Total number of shrubs >1 m height and trees <10 cm DBH from three quadrats (surrogate for availability of food, foraging substrates, shelter/nesting sites)
Stumps	Continuous	0–36	6.39	Yes	Yes	Total number of stumps from three quadrats (surrogate for availability of food and foraging substrates)
Logs	Continuous	0–15	2.87	Yes	Yes	Total number of logs ≥30 cm diameter from three quadrats (surrogate for availability of food and foraging substrates)
Group size^a^	Continuous	2–12	5.59	No	Yes	Average number of individuals in a territory
Neighbourhood size	Categorical	N/A	N/A	Yes	Yes	Large (>30 territories in population); small (≤30 territories) with ≥5 km between populations (indicator of habitat quality/availability at landscape-level)
Group isolation^a^	Continuous	0.00–4396.70	1008.80	Yes	Yes	Distance (m) from the nearest neighbour territory (indicator of local habitat fragmentation and exchange of individuals between groups)
Local tree cover^a^ ^b^	Continuous	0.41–19.04	7.25	Yes	Yes	Tree cover (ha) within a 300 m radius of where territories occurred (indicator of habitat available to each study group)
Landscape tree cover	Continuous	3.04–185.45	50.85	Yes	Yes	Tree cover (ha) within a 1 km radius of where territories occurred (indicator of local habitat fragmentation and exchange of individuals between groups)
Region^c^	Categorical	N/A	N/A	Yes	Yes	Three regions: west; south-east; north-east

^a^ Log_10_ transformed for breeding success models only

^b^ Range and mean values provided for untransformed data

^c^ Included as explanatory variable in generalised linear model for group size; included as a random term in all other models.

### 2.9 Model development and selection

Explanatory variables were grouped to represent distinct hypotheses regarding influences on breeding success and group size: (1) habitat structure variables (surrogates for food supply, shelter and nest sites); (2) local tree cover (local habitat availability); (3) territory isolation (local habitat fragmentation and exchange of individuals); and (4) local neighbourhood size (landscape-level habitat availability/quality). Group size was included as an additional hypothesis for the ‘fledgling occurrence’ response variable as it was likely to be an important predictor of breeding success. A single model was developed for each hypothesis group, and all possible combinations of groups were also assessed (total models: breeding success, *n* = 31; group size, *n* = 15). The nature of the relationship between response and explanatory variables was explored using scatterplots, component-and-residual plots, and the fit of simple univariate models. Explanatory variables (Table 1) were standardised for all generalised linear mixed-models and transformed to linearise relationships as required. Continuous explanatory variables were not highly correlated (*r* ≤0.4).

Hypotheses regarding breeding success (presence/absence of fledglings) were tested using generalised linear mixed-models employing a binomial error distribution. Study region (west, south-east, north-east) was included as a random term to account for possible non-independent error structures due to clustering of grey-crowned babbler territories [37]. This approach facilitates the identification of explanatory variables that influence the dependent variable irrespective of study region. Only sites where the age of all group members was identified throughout the 2010/11 breeding season were included in analyses of breeding success (*n* = 63).

Analyses of group size occurred in two stages. First, we were interested to know whether group size differed by region. A generalised linear model was constructed with region as the sole explanatory variable. Second, our four key hypotheses were tested using generalised linear mixed-models with region as a random term. A Poisson error distribution was employed in both cases. No overdispersion was detected in Poisson models.

Akaike’s information criterion corrected for small sample size (AIC_*c*_) was computed for all models representing the key hypotheses, as were AIC_*c*_ weights (*w*_*i*_; evidence in favour of a given model being the best of those considered) [38]. Akaike weights were summed (to a value of 0.95) to generate a 95% confidence set for the best model [38]. Model-averaging was conducted where no standout model (*w*_*i*_ >0.9) was identified [38]. Explanatory variables were considered to be an important influence on the dependent variable where the 95% confidence interval (standard error multiplied by 1.96) for model-averaged coefficients did not overlap zero. Model fit was assessed in various ways owing to the different modelling approaches used: (1) conditional and marginal pseudo-*R* ^*2*^ (binomial glmm; [39]); (2) adjusted D^*2*^ (Poisson glm; [6]); and (3) likelihood-ratio based pseudo-*R* ^*2*^ (Poisson glmm; [40]). Residual plots were inspected to ensure that model structures were appropriate for the data. All statistical analyses were performed in R ver. 3.0.2 [41] using the packages *car* ver. 2.0–19 [42], *lme4* ver. 1.0–5 [43], *AICcmodavg* ver. 1.35 [44], and *MuMIn* ver. 1.9.13 [40].

## Results

### 3.1 Comparison of habitat attributes within and between regions

Habitat attributes of territories differed between regions (ANOSIM Global R = 0.279; *p* = 0.001) (S2 Table) with the largest differences being between the west and the north-east (R = 0.417, *p* = 0.001), and the west and south-east (R = 0.364, *p* = 0.001). Habitat attributes did not differ between the south-east and north-east (R = 0.030, *p* = 0.112) (S1 Fig). The cover of short grass and leaf litter was a major contributor to regional differences, with reduced cover of these elements in territories in the west. The same variables contributed most to between-site similarities within regions (S3 Table).

### 3.2 Breeding success (fledgling presence/absence)

**Fig 2:**
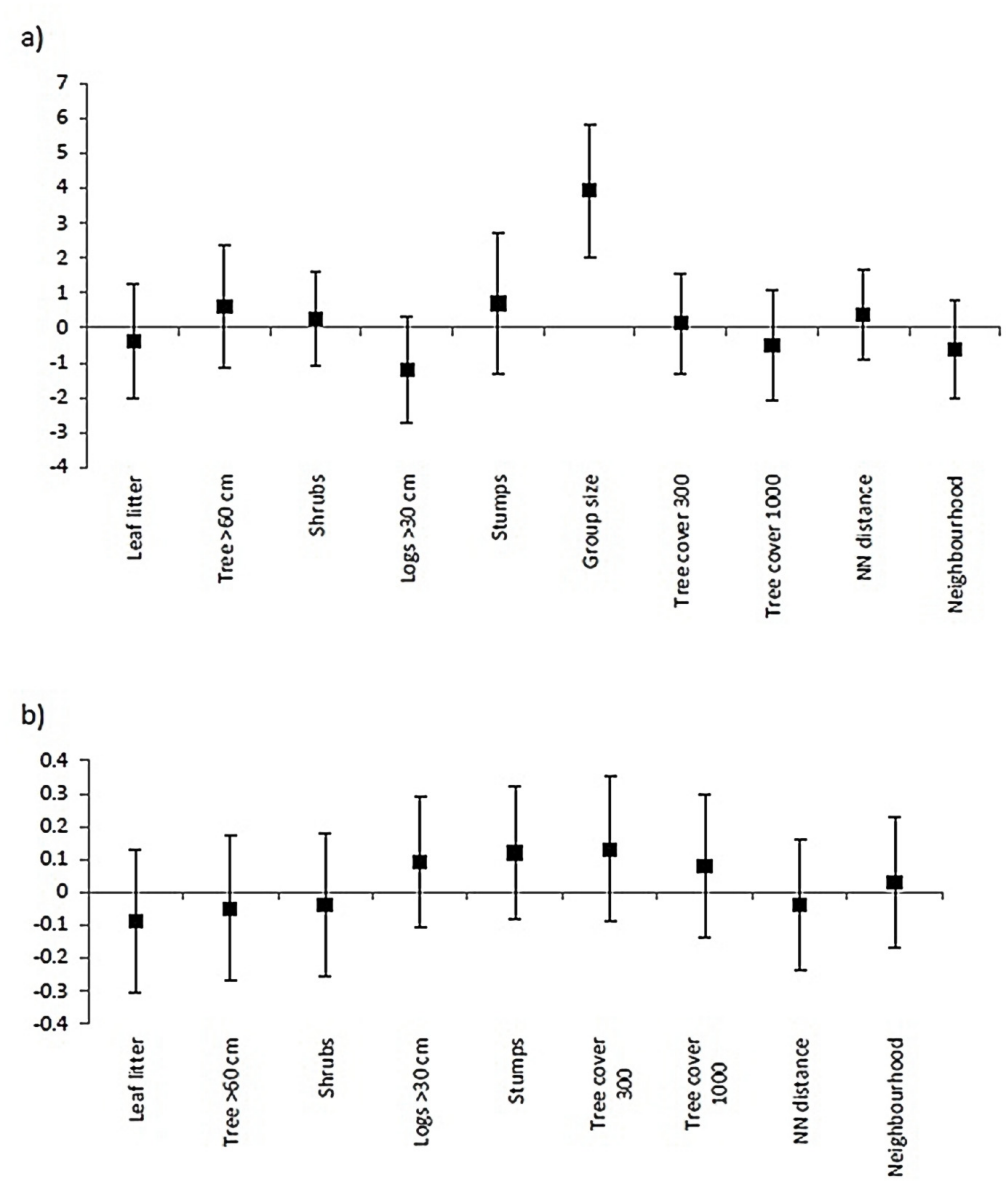
Model-averaged coefficients and associated 95% confidence intervals for explanatory variables included in models of (a) breeding success (fledgling presence/absence); and (b) group size of the grey-crowned babbler.

**Fig 3:**
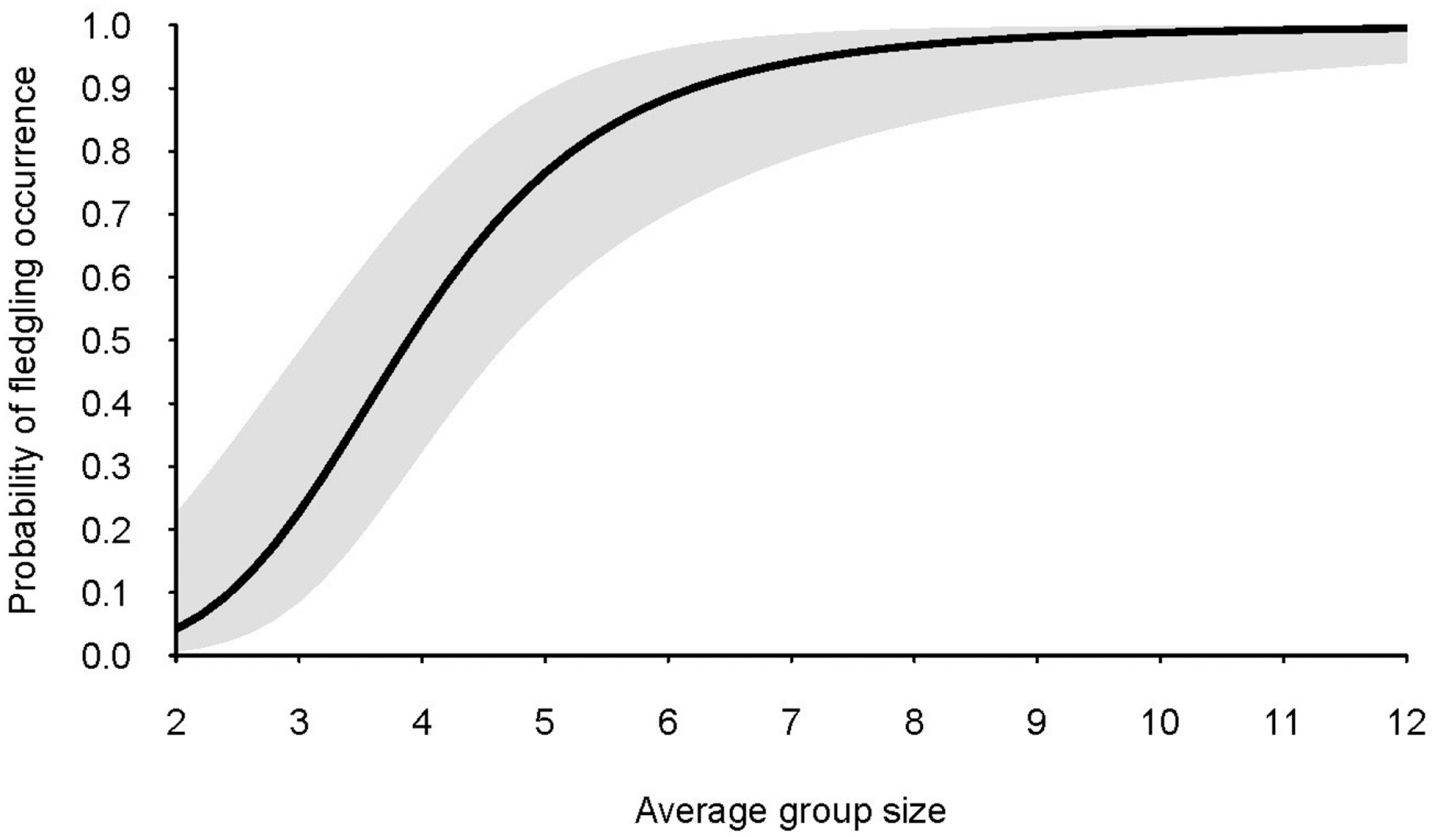
Predicted probability of occurrence of grey-crowned babbler fledglings as a function of average group size. Grey shading represents the 95% confidence interval for predicted values. Predictions were generated from model-averaged parameter estimates of generalized linear mixed models.

Two-thirds of monitored grey-crowned babbler groups produced fledglings during the 2010/11 breeding season. Of the models considered to account for fledgling occurrence, two had substantial support (Δ_*i*_ <2) (Table 2). Grey-crowned babbler group size was included in all five models in the 95% confidence set and was the single explanatory variable in the first ranked model, accounting for 54% of variance in the data (both marginal and conditional *R* ^*2*^ values = 54%). Model averaging revealed group size to be the only explanatory variable to have an important influence on the presence of fledglings (coefficient = 3.93; CI = 2.03, 5.83) (Fig 2a). The probability of occurrence of fledglings is predicted to increase rapidly as group size increases from two to seven (Fig 3). Once a group size of seven or more is attained, the probability of fledglings being detected exceeds 90%.

**Table 2:** Model-selection results for breeding success (fledgling presence/absence) and mean group size of the grey-crowned babbler (*Pomatostomus temporalis*).

Model name	Model components	df	*Log(l)*	AIC_*c*_	Δ_*i*_	*w*_*i*_
Breeding success	Group size	3	-26.2	58.8	0.0	0.4
	Group size & neighbourhood size	4	-25.8	60.2	1.5	0.2
	Group size & local tree cover	4	-26.0	61.0	2.3	0.1
	Group size & neighbourhood size & local tree cover	5	-25.8	62.6	3.8	0.1
	Group size & isolation	5	-25.8	62.7	3.9	0.1
Group size	Local tree cover	3	-156.3	319.0	0.0	0.4
	Neighbourhood size	3	-156.8	320.0	1.0	0.2
	Local tree cover & neighbourhood size	4	-156.3	321.2	2.2	0.1
	Group isolation	3	-156.4	321.5	2.4	0.1
	Local tree cover & group isolation	4	-156.2	323.4	4.4	0.0

The models shown are those within the 95% confidence set. Included are the number of parameters (df), log-likelihood values (*Log(l)*), AIC_*c*_ values, Akaike differences (Δ_*i*_), and Akaike weights (*w*_*i*_).

### 3.3 Group size

Of the 72 babbler groups monitored, group size ranged from 2–12 individuals (mean = 5.6) (S1 Table). Eight groups (11%) varied in group size during the study: four increased by 1–2 individuals while four decreased by 1–2 individuals.

Study region was an important influence on group size. Compared with the south-east region, group size was larger in the west (coefficient = 0.29; 95% CI = 0.05, 0.53). There was no difference in group size between the south-east and north-east regions (coefficient = 0.09; 95% CI = -0.17, 0.35). Average group size in the west was 6.5 (± 0.5 SE), while in the south-east it was 5.3 (± 0.4 SE) and in the north-east 4.9 (± 0.5 SE). The D^*2*^ value for this model was 7.0%, indicating that much variance remained unexplained.

Of the models considered, that relate our four key hypotheses to group size, two had substantial support (Δ_*i*_ <2), while the 95% confidence set comprised five models (Table 2). The first ranked model included only local tree cover and had a pseudo-*R* ^*2*^ value of 2.0%. Local tree cover occurred in three of the five models in the 95% confidence set (Table 2). However, model averaging indicated that none of the explanatory variables considered were an important influence on group size (i.e. 95% confidence intervals for all model-averaged coefficients overlapped zero) (Fig 2b).

## Discussion

While presence/absence studies provide a coarse measure of habitat suitability, studies of demographic parameters have the potential to reveal fine-scale variation in habitat quality across occupied sites. Here, we built on knowledge of core habitat preferences of the grey-crowned babbler derived from presence/absence studies to investigate whether there are particular attributes, at both site- and landscape-scales, associated with the quality of the species’ territories.

Overall, 66% of grey-crowned babbler groups were observed with one or more fledglings during the study. The probability of fledgling occurrence was positively correlated with group size. The number of available carers is known to positively influence reproductive success in a range of cooperatively breeding taxa (e.g. mammals: [45],birds: [46],fish: [47]) through enhancing food provisioning, parental and/or sentry duties, and predator defence [48–50]. The number of non-breeding helpers also has been shown to be important for the grey-crowned babbler, with larger groups having higher fledging success [18,19].

As a response variable, the size of grey-crowned babbler groups was influenced by study region, with group size being larger in the west compared to both the south-east and north-east regions. However, the vegetation attributes that differed most between territories in different regions (e.g. litter cover) were not useful predictors of group size and, controlling for regional effects, no site- or landscape-scale variables were found to influence group size. Agricultural land-use in the west is largely given to cropping, while in the two eastern regions there is both cropping and grazing. Fine-scale variation in landscape structure associated with variation in land-use, such as differential retention of paddock trees [51,52], may contribute to regional differences in group size.

The lack of stronger relationships between demographic parameters and site attributes is surprising, given that we chose attributes from a suite of variables previously hypothesized as being important indicators of territory quality for the grey-crowned babbler [16,17,53]. One explanation is that by surveying occupied sites only, spatial variation in key resources across studied territories may not have been sufficient to elicit consistent demographic change. Almost all territories were located in relatively open woodland vegetation and provided some large trees (>60 cm DBH), patchy tall shrubs or eucalypt regeneration, larger logs (>30 cm diameter), leaf litter and a sparse ground layer (Table 1). Grey-crowned babblers may not occupy sites where some or all of these characteristics are at low levels or absent [15,16]. Further, this species has declined markedly across this region [23] and it is possible that the species is now restricted largely to remaining areas of high quality habitat.

The number of grey-crowned babblers within a group is dynamic [18,20,54] and temporal changes in group size may obscure relationships with habitat attributes. Groups of more than 8–10 individuals are uncommon, with territory fission taking place as numbers increase, resulting in more groups of fewer individuals [54]. The size of a group also may decrease when offspring disperse or when a breeder dies and group disintegration occurs [54]. Conversely, group size may increase with successful reproduction [20] and with the immigration of individuals from nearby groups [54]. These dynamic processes may occur irrespective of the attributes of a site. Thus, it is plausible that groups of markedly different size may occupy territories of the same quality; in high quality territories a small group may be the result of recent fission, whilst a large group may be the result of recent reproductive success. Concurrent genetic research has identified an isolation-by-distance pattern amongst individuals in the study region, indicating that dispersal is locally restricted in fragmented habitat by between-group distances, and/or fragmentation effects. Thus, variation in group size may also be a consequence of restricted offspring dispersal.

Another possible reason for the lack of clear predictors of territory quality is that biotic interactions, such as competition and predation, may vary in strength across the study area and obscure relationships between breeding success, group size and environmental attributes. An aggressive interspecific competitor, the noisy miner (*Manorina melanocephala*) [55], was present at every grey-crowned babbler territory, and was observed to frequently harass and attack grey-crowned babblers as they foraged. We did not quantify the abundance of noisy miners at each territory, and so could not examine their influence on group size or reproductive success. However, the aggressive actions of this avian competitor, especially where they occur at higher densities, may influence the demography of the grey-crowned babbler. Avian predators of nests or fledglings, such as butcherbirds (*Cracticus* spp.), currawongs (*Strepera* spp.), ravens (*Corvus* spp.) and the laughing kookaburra (*Dacelo novaeguinea*), are common in small remnants and linear strips [56,57], and were regularly encountered at grey-crowned babbler territories during this study. Geographic variation in the abundance or impact of predators (e.g. more frequent occurrence of the pied currawong (*S*. *graculina*) in the east of our study area [58]), may also mask relationships between the demography of the grey-crowned babbler and indicators of habitat quality. The role of interspecific competition and predation as influences on the population performance of the grey-crowned babbler requires further research.

Our results highlight the importance of conservation actions that enhance the size of grey-crowned babbler family groups to facilitate higher probabilities of breeding success for this declining species. Several management projects within the study area show promise in achieving this goal [23,59]. They involve targeted restoration at a landscape scale with three main components: protecting and maintaining woodland remnants of known suitable habitat for the species (especially numerous large old trees); expanding the overall amount of wooded vegetation through complementary revegetation and regeneration; and increasing the connectivity of wooded habitats at the landscape scale [23,59]. Where such work has been undertaken there is evidence that the number of grey-crowned babbler groups has either stabilised or increased, and that group size is dynamic over time. Long-term monitoring of family groups and group size dynamics, in tandem with an assessment of the impacts of competition and predation, will be of great value in more clearly pinpointing the environmental attributes that indicate territory quality and enhanced breeding success.

Studies of population demography typically are more labour intensive and time consuming than presence/absence studies. Strong relationships between demographic parameters and explanatory variables can also be more difficult to detect, especially when only occupied sites are surveyed. However, studies of population processes can lead to the development of new hypotheses and research questions, as in this study. Demographic studies have the potential to identify the mechanistic processes that underpin population performance in human modified landscapes, and knowledge of such processes is critical for effective conservation management.

## Supporting Information

S1 Table Group size of the grey-crowned babbler and the number of groups that recorded breeding success at least once during the period June 2010 to April 2011, for study sites across the west, south-east and north-east study regions. (PDF)

S2 Table Comparison between regions of habitat characteristics in grey-crowned babbler territories based on ANOSIM. (PDF)

S3 Table Within-region similarities in habitat characteristics of grey-crowned babbler territories. (PDF)

S1 Fig Non-metric multi-dimensional scaling ordination of study sites within three regions based on habitat attributes. (PDF)
