# Supplementary material for: What Determines Habitat Quality for a Declining Woodland Bird in a Fragmented Environment: The Grey-Crowned Babbler Pomatostomus temporalis in South-Eastern Australia?
Source: PLoS One. 2015 Jun 22;10(6):e0130738. doi: 10.1371/journal.pone.0130738 (PMC4476705; doi:10.1371/journal.pone.0130738)
Supplement: S1 Table — (PDF) [file pone.0130738.s001.pdf]

S1 Table

S1 Table. Group size of the Grey-crowned Babbler and the number of groups that recorded breeding success at least once during the period June 2010 to April 2011, for study sites across the west, south-east and north-east study regions. Percentages are shown in parentheses.

Region	Group size			Total individuals	No. of groups	No. of groups detected with fledglings (%)
	Min	Max	Mean			
West	3	12	6.6	158	24	16 (67)
South-east	2	9	5.4	130	24	14 (52)
North-east	2	12	4.9	117	24	12 (50)
Total	2	12	5.6	405	72	42 (58)
