# Supplementary material for: What Determines Habitat Quality for a Declining Woodland Bird in a Fragmented Environment: The Grey-Crowned Babbler Pomatostomus temporalis in South-Eastern Australia?
Source: PLoS One. 2015 Jun 22;10(6):e0130738. doi: 10.1371/journal.pone.0130738 (PMC4476705; doi:10.1371/journal.pone.0130738)
Supplement: S2 Table — (PDF) [file pone.0130738.s002.pdf]

S2 Table

S2 Table. Comparison between regions of habitat characteristics in grey-crowned babbler territories based on ANOSIM: (i) west (w) versus south-east (Se) (ii) west versus north-east (Ne), (iii) south-east versus north-east. The table shows variables that contributed to 90% of the dissimilarity between regions (average dissimilarity for (i) W v Se = 34.8; (ii) W v Ne = 36.6; (iii) Se v Ne = 24.6). The largest regional variable cover in each comparison is shown in bold.

		Mean cover (%) within region					
Regions	Variable	West	South-east	North-east	mean dissimilarity	SD	contribution %
(i) W v Se	Grass (short)	31.2	65.2		12.0	1.9	34.4
	Leaf litter	**61.5**	**72.8**		6.7	1.3	19.2
	Shrub	13.6	9.0		5.3	0.8	15.2
	Grass (tall)	2.7	5.8		1.6	1.3	4.7
	Tree (< 10 cm DBH)	7.2	9.1		2.9	0.9	8.3
	Tree (10-30 cm DBH)	7.8	9.1		2.3	1.3	6.5
	Lignum	4.9	0.0		1.6	0.9	4.4
(ii) W v Ne	Grass (short)	31.2	X	**69.9**	13.4	2.00	36.6
	Leaf litter	**61.5**	X	66.8	6.4	1.3	17.4
	Shrub	13.6	X	3.1	4.5	0.7	12.4
	Grass (tall)	2.7	X	13.3	3.7	1.3	10.1
	Tree (< 10 cm DBH)	7.2	X	6.9	2.5	0.8	6.9
	Tree (10-30 cm DBH)	7.8	X	6.4	2.2	1.3	6.1
	Lignum	4.9	X	0.0	1.6	0.9	4.2
(iii) Se v Ne	Grass (short)	X	65.2	**69.7**	7.4	1.2	29.9
	Leaf litter	X	**72.8**	66.8	5.4	1.2	22.1
	Shrub	X	9.0	3.1	2.7	0.5	10.9
	Grass (tall)	X	5.8	13.3	2.8	1.1	11.2
	Tree (< 10 cm DBH)	X	9.1	6.9	2.4	0.8	9.8
	Tree (10-30 cm DBH)	X	9.1	6.4	1.9	1.3	7.8
