# Supplementary material for: What Determines Habitat Quality for a Declining Woodland Bird in a Fragmented Environment: The Grey-Crowned Babbler Pomatostomus temporalis in South-Eastern Australia?
Source: PLoS One. 2015 Jun 22;10(6):e0130738. doi: 10.1371/journal.pone.0130738 (PMC4476705; doi:10.1371/journal.pone.0130738)
Supplement: S3 Table — (PDF) [file pone.0130738.s003.pdf]

S3 Table

S3 Table. Within-region similarities in habitat characteristics of grey-crowned babbler territories. The table shows variables that contributed to 90% of similarity within regions

Region	Habitat variable	Average abundance	Average similarity	Similarity SD	Contribution %	Cumulative %
West<br>Avg. similarity: 65.81	Leaf litter (%)	61.50	37.74	4.13	57.36	57.36
	Grass < 10 cm ht (%)	31.24	16.02	1.91	24.35	81.71
	Tree 10 - 30 cm DBH	7.84	2.75	1.02	4.17	85.88
	Shrub > 1 m ht	13.63	2.32	0.41	3.53	89.41
	Tree < 10 cm DBH	7.15	1.94	0.85	2.96	92.36
North-east<br>Avg. similarity: 76.84	Grass < 10 cm ht (%)	69.86	33.09	3.21	43.06	43.06
	Leaf litter (%)	66.84	32.91	4.53	42.83	85.89
	Grass 40 cm ht (%)	13.28	4.69	1.49	6.11	92.00
South-east<br>Avg. similarity: 75.10	Leaf litter (%)	72.80	36.13	5.15	48.11	48.11
	Grass < 10 cm ht (%)	65.18	28.46	3.00	37.90	86.00
	Tree 10 - 30 cm DBH	9.10	3.41	1.67	4.55	90.55
