# Supplementary material for: What Determines Habitat Quality for a Declining Woodland Bird in a Fragmented Environment: The Grey-Crowned Babbler Pomatostomus temporalis in South-Eastern Australia?
Source: PLoS One. 2015 Jun 22;10(6):e0130738. doi: 10.1371/journal.pone.0130738 (PMC4476705; doi:10.1371/journal.pone.0130738)

S1 Fig.

S1 Fig. Non-metric multi-dimensional scaling ordination of study sites within three regions based on the differences in habitat attributes. West region = black triangles; south-east region = grey circles; north-east region = white diamonds

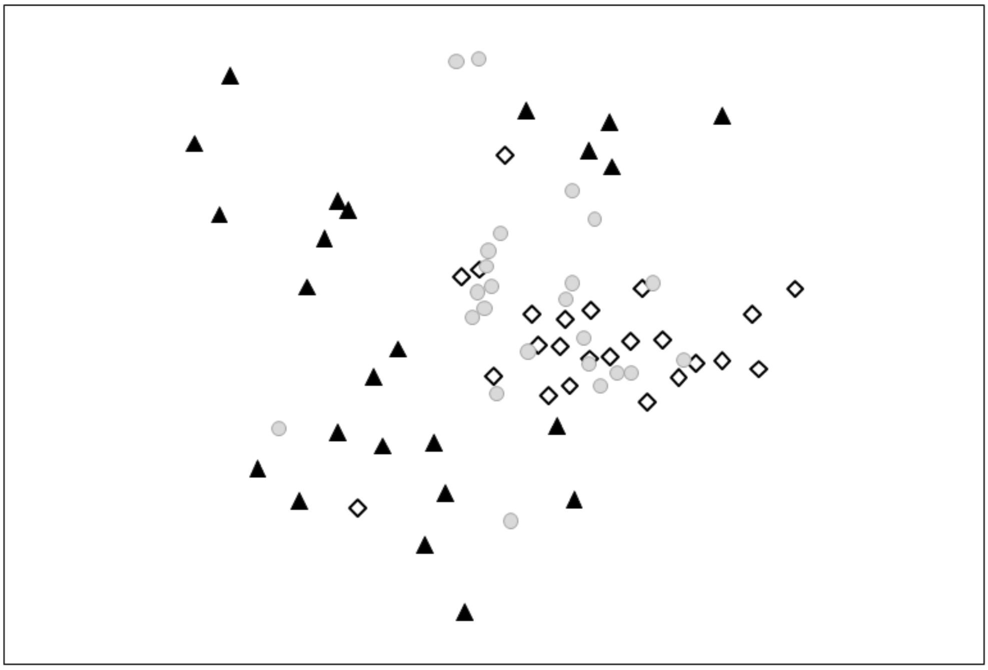

Supplement: S1 Fig — (PDF) [file pone.0130738.s004.pdf]
